# Effects of *Schyzocotyle acheilognathi* (Yamaguti, 1934) infection on the intestinal microbiota, growth and immune reactions of grass carp (*Ctenopharyngodon idella*)

**DOI:** 10.1371/journal.pone.0266766

**Published:** 2022-04-12

**Authors:** Pei P. Fu, Fan Xiong, Shan G. Wu, Hong Zou, Ming Li, Gui T. Wang, Wen X. Li

**Affiliations:** 1 Key Laboratory of Aquaculture Disease Control, Ministry of Agriculture, and State Key Laboratory of Freshwater Ecology and Biotechnology, Institute of Hydrobiology, Chinese Academy of Sciences, Wuhan, P. R. China; 2 University of Chinese Academy of Sciences, Beijing, P. R. China; 3 Center for Energy Metabolism and Reproduction, Institute of Biomedicine and Biotechnology, Shenzhen Institute of Advanced Technology, Chinese Academy of Sciences, Shenzhen, Guangdong, P. R. China; University of Illinois Urbana-Champaign, UNITED STATES

## Abstract

Our understanding of interactions among intestinal helminths, gut microbiota and host is still in its infancy in fish. In this study, the effects of *Schyzocotyle acheilognathi* infection on the intestinal microbiota, growth and immune reactions of grass carp were explored under laboratory conditions. 16S rDNA amplification sequencing results showed that *S*. *acheilognathi* infection altered the composition of intestinal microbiota only at the genus level, with a significant increase in the relative abundance of *Turicibacter* and *Ruminococcus* (*P* < 0.05) and a significant decrease in the relative abundance of *Gordonia*, *Mycobacterium* and *Pseudocanthomonas* (*P* < 0.05). *Schyzocotyle acheilognathi* infection had no significant effect (*P* > 0.05) on the alpha diversity indices (including Chao1, ACE, Shannon, Simpson index) of intestinal microbiota in grass carp, but PERMANOVA analysis showed that microbial structure significantly (*P* < 0.01) differed between hindgut and foregut. PICRUST prediction showed that some metabolism-related pathways were significantly changed after *S*. *acheilognathi* infection. The relative abundance of *Turicibacter* was positively correlated with the fresh weight of tapeworm (foregut: *r* = 0.48, *P* = 0.044; hindgut: *r* = 0.63, *P* = 0.005). There was no significant difference in the body condition of grass carp between the *S*. *acheilognathi* infected group and the uninfected group (*P* > 0.05). Intestinal tissue section with HE staining showed that *S*. *acheilognathi* infection severely damaged the intestinal villi, causing serious degeneration, necrosis and shedding of intestinal epithelial cells. The real-time fluorescent quantitative PCR results showed that *S*. *acheilognathi* infection upregulated the mRNA expression of the immune-related genes: *Gal1*−*L2*, *TGF*−*β1* and *IgM*.

## Introduction

Vertebrates’ gastrointestinal (GI) tract harbors a dynamic and complicated micro-ecosystem, including bacteria, fungi, protozoan and helminth parasites. They co-evolve with the host, and they are vital to the host’s physiology and homeostasis [1]. Sharing the same niche in the host intestine, microbiota and helminths can interact with each other [2].

Increasing evidence, particularly in humans and rodent models of helminth infection experiments, indicates that a multitude of interactions occurs between parasites and gut microbiota [3, 4]. Many studies have shown that the composition and diversity of vertebrates’ gut microbiota can be altered by the hosts’ helminth parasites [1]: some species can increase [5, 6] or reduce [7–9] host’ intestinal microbiota diversity. Contrary to this, in some cases, helminths infections do not affect the intestinal microbiota diversity [10–14]. But even in those cases, the composition of microbiota may be altered. Moreover, the long-term existence of helminths in the GI tract also depends on host’s gut microbiota. For example, *Trichuris muris* infection selected for a distinct intestinal microbiota profile [4]. This, in turn, affected the fitness of this nematode parasite, as reducing the number of bacteria in the host significantly reduced the number of hatched *T*. *muris* eggs [3]. Administration of live or dead *Lactobacillus casei* to mice enhanced susceptibility to *T*. *muris* [15]. Similarly, *H*. *polygyrus* was less able to form persistent infections in germ-free mice (lacking microbiota) compared with conventionally raised mice [16]. Administration of *L*. *taiwanensis* significantly enhanced *H*. *polygyrus* burden and prolonged the persistence of infection [17].

Host metabolism is largely dependent on the gut microbiota. Thus, helminth-induced changes to the microbiota inevitably modify the host’s metabolism. Helminth infection can modify the metabolic capacity of the mammalian hosts [18]: several studies indicated that *Trichuris* infection reduced carbohydrate metabolism [6, 19, 20] or a caused reduction in the breakdown products of plant-derived carbohydrates [8]. Short-chain fatty acids (SCFAs) are largely derived from the bacterial fermentation of complex oligosaccharides present in the diet [21]. Helminth infections increase SCFAs concentration via altering gut microbiota [22, 23].

Meanwhile, the helminth-modified intestinal microbiota has the capacity to modify host immune response [18]. For example, transferring the fecal microbiota (FMT) from helminth-infected mice can ameliorate allergic airway inflammation [22]. However, there are only sporadic studies about interactions between GI helminths and fish gut microbiota. The few previously conducted studies have been limited to the impact of helminths on the composition of intestinal microbiota [24–26], but the tripartite interaction among helminths, intestinal microbiota and host has not yet been studied in fish.

Grass carp (*Ctenopharyngodon idella*) is one of the most important economic freshwater fish species in China, where the production reached 5.5 million tons in 2019 and constitutes 21.6% of the total freshwater-cultured fish annual output. GI helminth *Schyzocotyle acheilognathi* Yamaguti, 1934 (*syn*. *Bothriocephalus acheilognathi*) is one of the most harmful pathogens to grass carp [27, 28]. It mainly infects one to two-years-old grass carp fry. The grass carp severely infected with *S*. *acheilognathi* will lose weight, even become emaciated, and large numbers of tapeworms can even cause the death of the host [27]. Tapeworms are parasitic Platyhelminthes with no digestive tract. It is generally believed that it absorbs nutrients from the host’s intestinal tract by the specialized microtriches of the tegument [29]. Tapeworms attach to the intestinal wall of the host by the bothrium of the scolex, causing local inflammation and pathological changes [30], while the strobilus is free in the intestinal cavity. It remains unknown how does the tapeworm absorbs large granules of cellulose in the intestine of grass carp and causes mass death of the host, but we hypothesize that this may be closely related to the gut microbiota. Exploring the effects of *S*. *acheilognathi* infection on intestinal microbiota, growth and immune reactions of grass carp will lay a foundation for elucidating the tripartite interaction among helminths, intestinal microbiota and host in fish.

## Materials and methods

### Ethics statement

All animal experiments complied with the ARRIVE guidelines and carried out in accordance with the National Institutes of Health guide for the care and use of laboratory animals (NIH Publications No. 8023, revised 1978). All surgeries were performed under MS-222 (final concentration: 50 mg/L) and all efforts were made to alleviate suffering. All protocols were approved by the committee of the Institute of Hydrobiology, Chinese Academy of Sciences (CAS). The reference number obtained was Y11201-1-301 (Approval date: 30 May 2016).

### Grass carp culture and sample collection

The fry of grass carp (9.2 ± 0.68 cm) was raised in an aquaculture pond in Guangzhou, Guangdong province. A preliminary investigation found that there was a high incidence of *S*. *acheilognathi* infection in grass carp in the pond. The grass carp was temporarily kept in the laboratory for three days before the formal experiment. The experiment lasted for 15 days, and during the experimental period, fish were kept in circulating-water aquariums under the natural photoperiod conditions (12h: 12h), water temperature ranged from 25 to 26 °C, and pH fluctuated from 7.2 to 7.4. The fish were fed to apparent satiation twice a day (9:00, 18:00 o’clock).

Grass carp (*n* = 51) were anesthetized with MS-222 (50 mg/L) and then the intestinal tracts were aseptically removed from the abdominal cavity. The samples were divided into the infected group and uninfected group according to whether the specimen was infected with *S*. *acheilognathi* or not. Intestinal content (foregut and hindgut) was collected for bacterial 16S rDNA sequencing, and the foregut was frozen immediately in liquid nitrogen and stored at −80 °C until RNA extraction. The foregut was fixed with 4% paraformaldehyde solution for intestinal histology observation. After removing it from the foregut, *S*. *acheilognathi* was placed in sterile PBS to wash off the surface contents of the intestine, and tapeworm wet weight was recorded. Finally, tapeworms were frozen in liquid nitrogen, and later used for bacterial 16S rDNA sequencing.

### The total bacterial DNA extraction, 16S rDNA amplification, and Illumina high throughput sequencing

The total bacterial DNA was extracted using QIAamp^®^ DNA stool mini kit (Qiagen, New York, USA) according to the manufacturer’s instructions. The purity and concentration of genomic DNA were determined with a spectrophotometer (Nanodrop 8000; Thermo Fisher Scientific, Wilmington, USA). DNA was stored at −20 °C for later use.

The universal primer pair 338F (5′- ACT CCT ACG GGA GGC AGC AG—3′) and 806R (5′- GGA CTA CHV GGG TWT CTA AT-3′) was used to amplify the V3-V4 hypervariable region of the bacterial 16S rDNA gene [31]. The PCR amplification program was the same as previously reported [24]. PCR products were subjected to electrophoresis, and the correct band (about 460 bp) was recovered using AidQuick Gel Extraction Kit (Aidlad Biotech, Beijing, China). The DNA concentration and purity were determined by a spectrophotometer (Nanodrop 8000). Sequencing was conducted by the Majorbio company (Shanghai, China) using the Illumina MiSeq PE300 platform. The obtained raw 16S rRNA sequences are available in the NCBI SRA database (Bioproject: PRJNA755354).

### Sequence data processing and analysis

The raw sequenced data were processed as described in Fu et al. (2019) [24]. Non-chimera sequences were firstly subsampled to the same sequence depth (31,790 reads per sample) using daisychopper.pl, then it was clustered into Operational taxonomic units (OTUs) at 97% similarity level using CD-HIT [32]. Singletons were filtered out. OTUs were annotated with Greengenes database (release 13.8) [33] using UCLUST. Sequences classified as unassigned and C_Chloroplast were removed.

Alpha diversity (Chao1, ACE, Shannon and Simpson index) and beta diversity (weighted unifrac metric and Bray-Curtis distance) indices of bacterial communities were calculated. Cluster analysis was performed on Bray-Curtis distance matrices of bacterial OTUs using an unweighted pair group mean algorithm (UPGMA). Principal coordinate analysis (PCoA) was used to visualize similarities between groups with weighted unifrac distance. PERMANOVA analysis was performed to test for significant differences between groups in overall microbial composition with weighted unifrac distance applying the Vegan package in R. Pearson’s correlation coefficient was used to investigate the degree of linear correlation between the wet weight of tapeworms and the abundance of bacteria using PAST 2.16. A Venn diagram of shared and unique OTUs was used to describe the similarities and differences of groups. Linear discriminant analysis coupled with effect size (Lefse) was used to study the significance of species differences at the genus level. The metagenomic content of samples was inferred from 16S rDNA gene sequence data using PICRUST 1.0 and KEGG database [34]. Stamp v2.1.3 was used for all statistical analyses of functional profiles [35].

### RNA isolation and real-time quantitative PCR

Total RNAs were extracted using TRIzol reagent. Two μg of total RNA treated with RNase-free DNase I (Promega, Wisconsin, USA) was used for synthesizing the first-strand cDNAs by ReverTraAce kit (Toyobo, Osaka, Japan) and oligo (dT) primers in 20 μL reaction solution. RT-qPCR was carried out using iQ^™^ SYBR Green Supermix (BioRad, Hercules, CA, USA) on a CFX96^™^ Real Time Detection System (BioRad). Pairs of gene-specific primers (Table 1) were used to amplify fragments of immune-related genes fragment. The *β-actin* of grass carp (Accession No. M25013.1) was selected as internal control and amplified with specific primers (Table 1). The RT-qPCR cycling conditions were as follows: 95 °C for 2 min, 40 cycles of 95 °C for 10 s, annealing at 62 °C for 20 s, and 72 °C for 30 s, followed by a Melt Curve analysis. Finally, the Ct values for respective reactions were inferred using the comparative Ct method (2^−ΔΔCT^) [36] to calculate the relative expression of immune-related genes in the intestinal mucosa. Statistical analysis was conducted using Student’s t-test by SPSS 16.0 at the 0.05 significance threshold.

**Table 1:** Primers used for RT-qPCR.

Primers	Sequences (5′-3′)
qβ-actin-F	TCGGTATGGGACAGAAGGAC
qβ-actin-R	GACCAGAGGCATACAGGGAC
Gal1-L2-F	GCCCATGGTGACCACCACACT
Gal1-L2-R	TCAGCACCTTGACGGTTAGGGA
qIL-4-F	CTACTGCTCGCTTTCGCTGT
qIL-4-R	CCCAGTTTTCAGTTCTCTCAGG
qIL-10-F	TATTAAACGAGAACGTGCAACAGAA
qIL-10-R	TCCCGCTTGAGATCTTGAAATATACT
qIFN-γ-F	CCAAAAGCGAGATGACCCA
qIFN-γ-R	CAAGCAACAGCGCCTGAC
qTGF-β1-F	CCACTGTAGAACTAAACCAGGAG
qTGF-β1-R	CTGTGATGTTGAACCATATGTGC
qIgM-F	GCTGAGGCATCGGAGGCACAT
qIgM-R	TTGGGTCTCGCACCATTTTCTC

### Intestinal histology of grass carp

The foregut of grass carp infected or uninfected with *S*. *acheilognathi* was used for histological observations. Standard protocols of intestinal sections are as follows: embedding the samples in paraplast, sectioning by microtome, staining with hematoxylin and eosin, and mounting in Canada balsam [37]. Measurements were in micrometers (μm).

## Results

### Microbiota composition in the intestine of grass carp and *S*. *acheilognathi* surface

We performed 16S rDNA sequencing on foregut and hindgut contents of grass carp uninfected or infected with tapeworms. We also sequenced microbiota on the surface of *S*. *acheilognathi*. Thus, we divided samples into five groups: UnF (uninfected with *S*. *acheilognathi* in the foregut of grass carp, *n* = 9), UnH (uninfected with *S*. *acheilognathi* in the hindgut of grass carp, *n* = 9), C (*S*. *acheilognathi* surface, *n* = 9), InF (infected with *S*. *acheilognathi* in the foregut of grass carp, *n* = 9), and InH (infected with *S*. *acheilognathi* in the hindgut of grass carp, *n* = 9).

**Fig 1:**
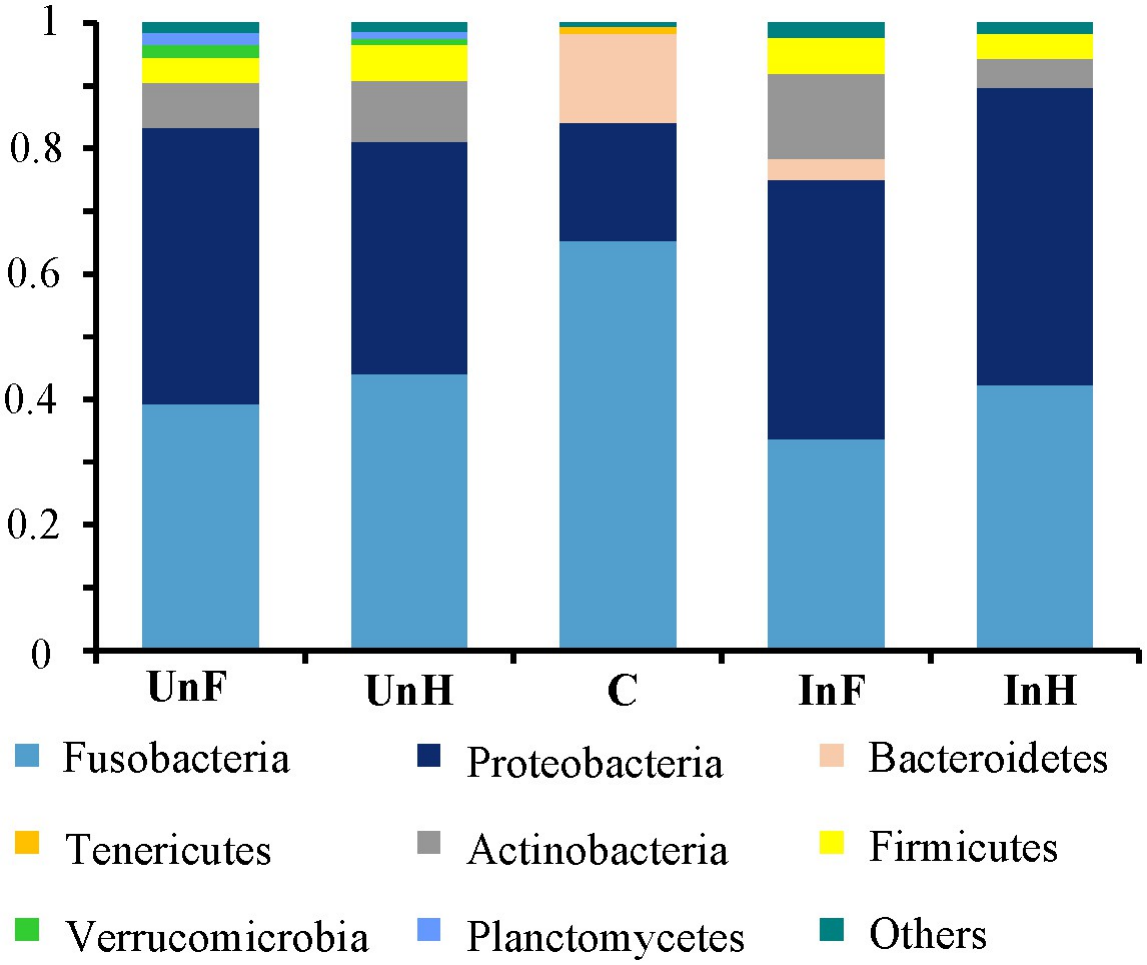
Microbiota composition in foregut and hindgut of grass carp and *S*. *acheilognathi* surface at the phylum level. UnF, uninfected with *S*. *acheilognathi* in the foregut of grass carp; UnH, uninfected with *S*. *acheilognathi* in the hindgut of grass carp; C, the surface of *S*. *acheilognathi*; InF, infected with *S*. *acheilognathi* in the foregut of grass carp; InH, infected with *S*. *acheilognathi* in the hindgut of grass carp; Others, the sum of different taxa with an abundance less than 1% in the samples.

Microbiota composition differed between the intestine of grass carp and *S*. *acheilognathi* surface. At the phylum level (Fig 1), the grass carp intestine held a core microbiota composed of Fusobacteria, Proteobacteria, Actinobacteria and Firmicutes. However, Fusobacteria, Proteobacteria, Bacteroidetes and Tenericutes were the dominant taxa on the surface of *S*. *acheilognathi*.

Compared with the UnF group, the relative abundance of Fusobacteria (39.28 ± 28.06% *vs*. 33.68 ± 37.81%), Proteobacteria (44.0 ± 22.61% *vs*. 41.31 ± 21.56%), Verrucomicrobia (2.05 ± 4.78% *vs*. 0.61 ± 0.69%) and Planctomycetes (1.81 ± 2.23% *vs*. 0.47 ± 0.28%) in the InF was lower, but differences were not significant (*P* > 0.05 in all cases). The relative abundance of Bacteroidetes (0.58 ± 0.82% *vs*. 3.45 ± 5.78%), Actinobacteria (7.22 ± 4.31% *vs*. 13.53 ± 12.10%) and Firmicutes (4.01 ± 5.40% *vs*. 5.61 ± 6.04%) in the InF increased, but difference in these taxa between the two groups were also not significant (*P* > 0.05 in all cases). However, compared with the UnH group, the relative abundance of Fusobacteria (44.17 ± 33.73% *vs*. 42.39 ± 31.56%), Actinobacteria (9.67 ± 6.29% *vs*. 4.66 ± 3.54%), Firmicutes (5.72 ± 7.74% *vs*. 4.03 ± 2.44%), Verrucomicrobia (1.03 ± 1.54% *vs*. 0.29 ± 0.71%) and Planctomycetes (1.09 ± 2.13% *vs*. 0.24 ± 0.48%) in the InH group non-significantly (*P* > 0.05 in all cases) decreased. The relative abundance of Proteobacteria (37.03 ± 22.13% *vs*. 47.26 ± 29.74%) in the InF group non-significantly increased (*P* > 0.05).

**Fig 2:**
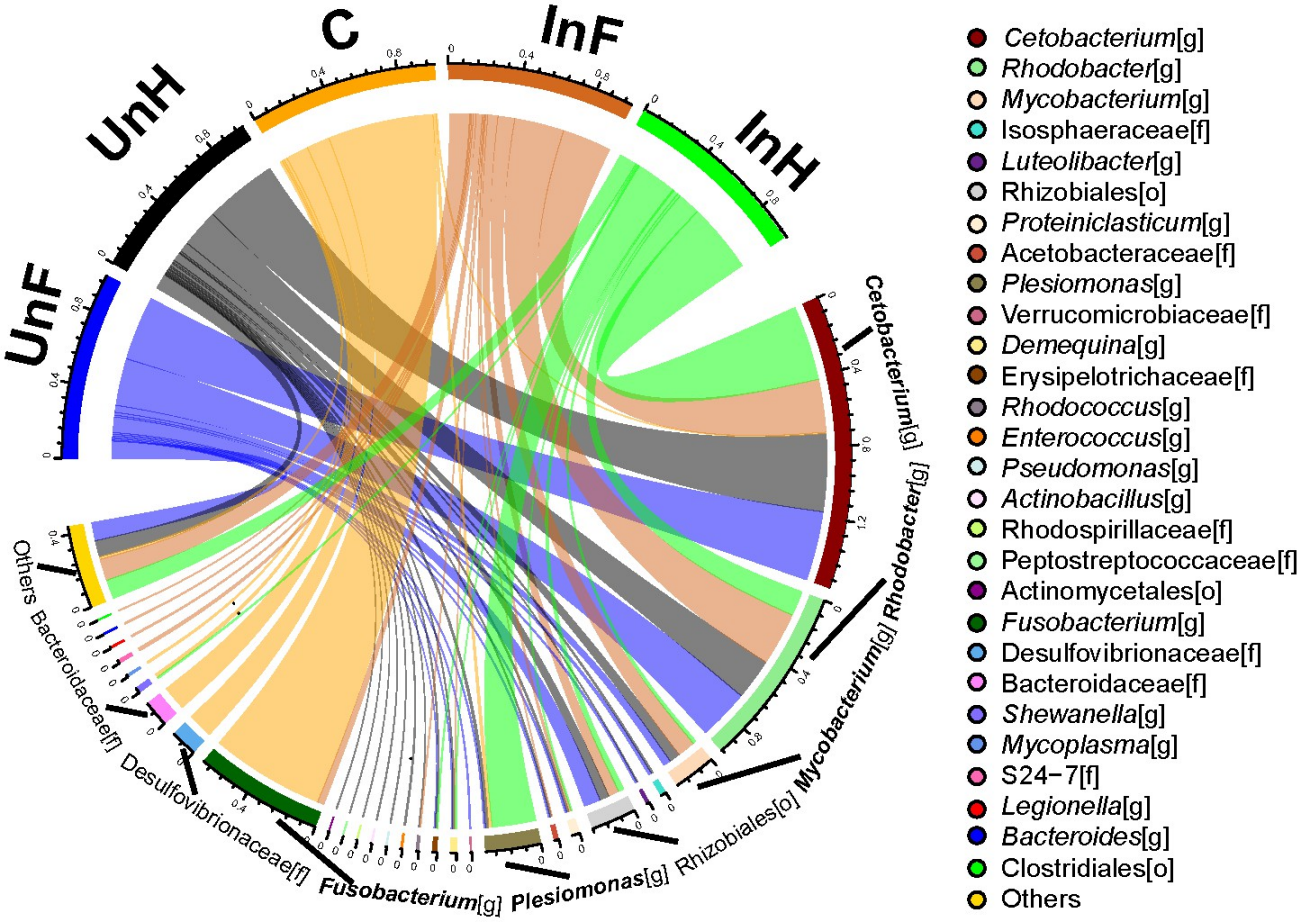
Microbiota composition in the foregut and hindgut of grass carp and on *S*. *acheilognathi* surface at the genus level. UnF, uninfected with *S*. *acheilognathi* in foregut of grass carp; UnH, uninfected with *S*. *acheilognathi* in the hindgut of grass carp; C, the surface of *S*. *acheilognathi*; InF, infected with *S*. *acheilognathi* in the foregut of grass carp; InH, infected with *S*. *acheilognathi* in the hindgut of grass carp; Others: the sum of different taxa with an abundance less than 0.5% in the samples.

At the genus level, *Cetobacterium*, *Rhodobacter*, Rhizobiales and *Mycobacterium* were the dominant taxa in the intestine of grass carp (Fig 2). *Fusobacterium*, Desulfovibrionaceae, Bacteroidaceae, *Shewanella* and *Mycoplasma* were the dominant taxa on the *S*. *acheilognathi* surface.

### Diversity of microbiota in the intestine of grass carp and *S*. *acheilognathi* surface

**Fig 3:**
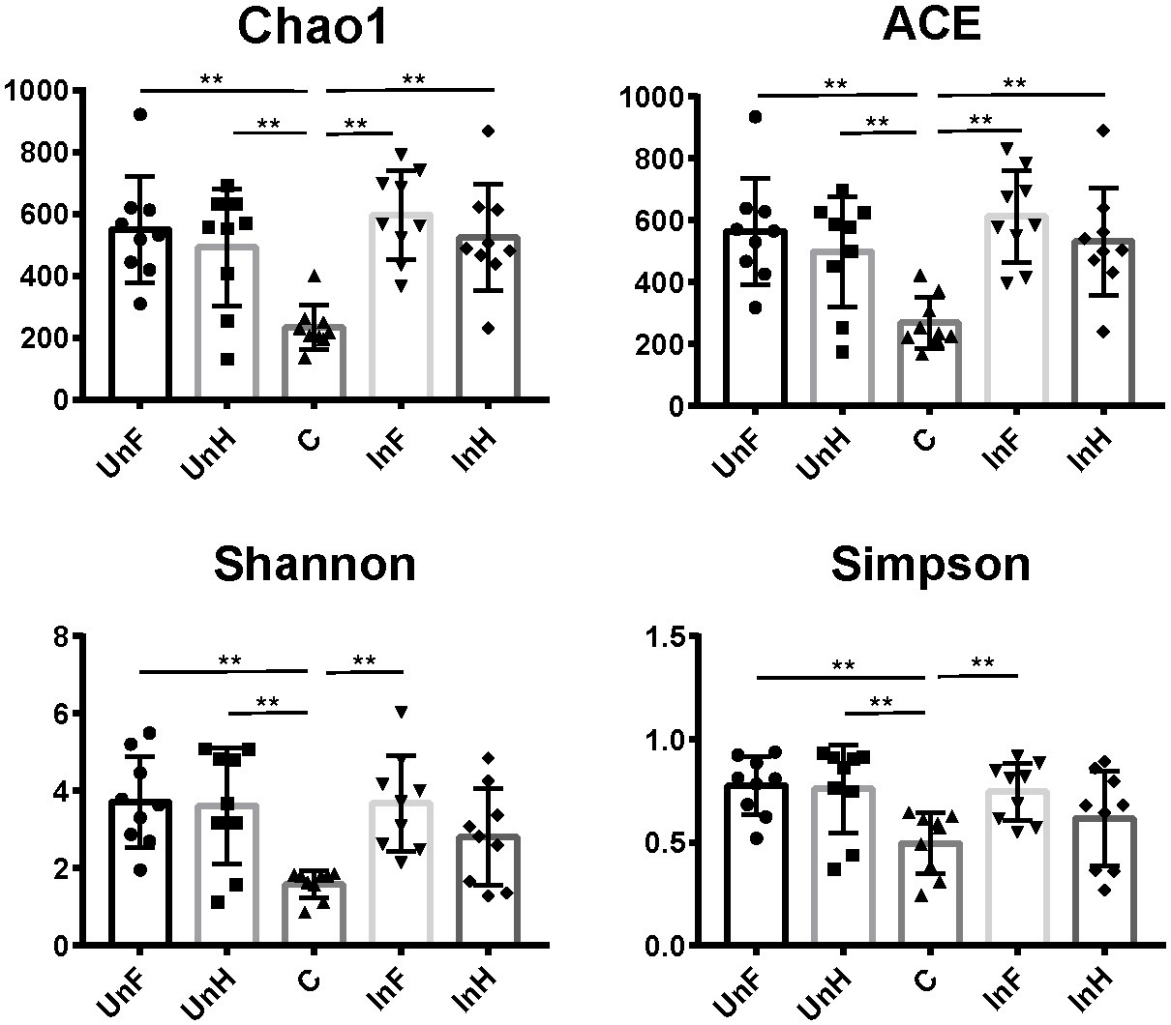
Alpha diversity indices of the gut microbiota of grass carp and on the surface of *S*. *acheilognathi*. UnF, uninfected with *S*. *acheilognathi* in the foregut of grass carp; UnH, uninfected with *S*. *acheilognathi* in the hindgut of grass carp; C, the surface of *S*. *acheilognathi*; InF, infected with *S*. *acheilognathi* in the foregut of grass carp; InH, infected with *S*. *acheilognathi* in the hindgut of grass carp.

Alpha diversity of microbiota on the tapeworm surface was mostly significantly lower thanin the grass carp intestine (*P* < 0.05 in all cases, except for Shannon: C and InH, Simpson: C and InH). Microbiota in the foregut of grass carp exhibited a slightly higher alpha diversity than in the hindgut, but there were no significant differences between UnF and InF or UnH and InH in alpha diversity (Fig 3, *P* > 0.05 in all cases).

**Fig 4:**
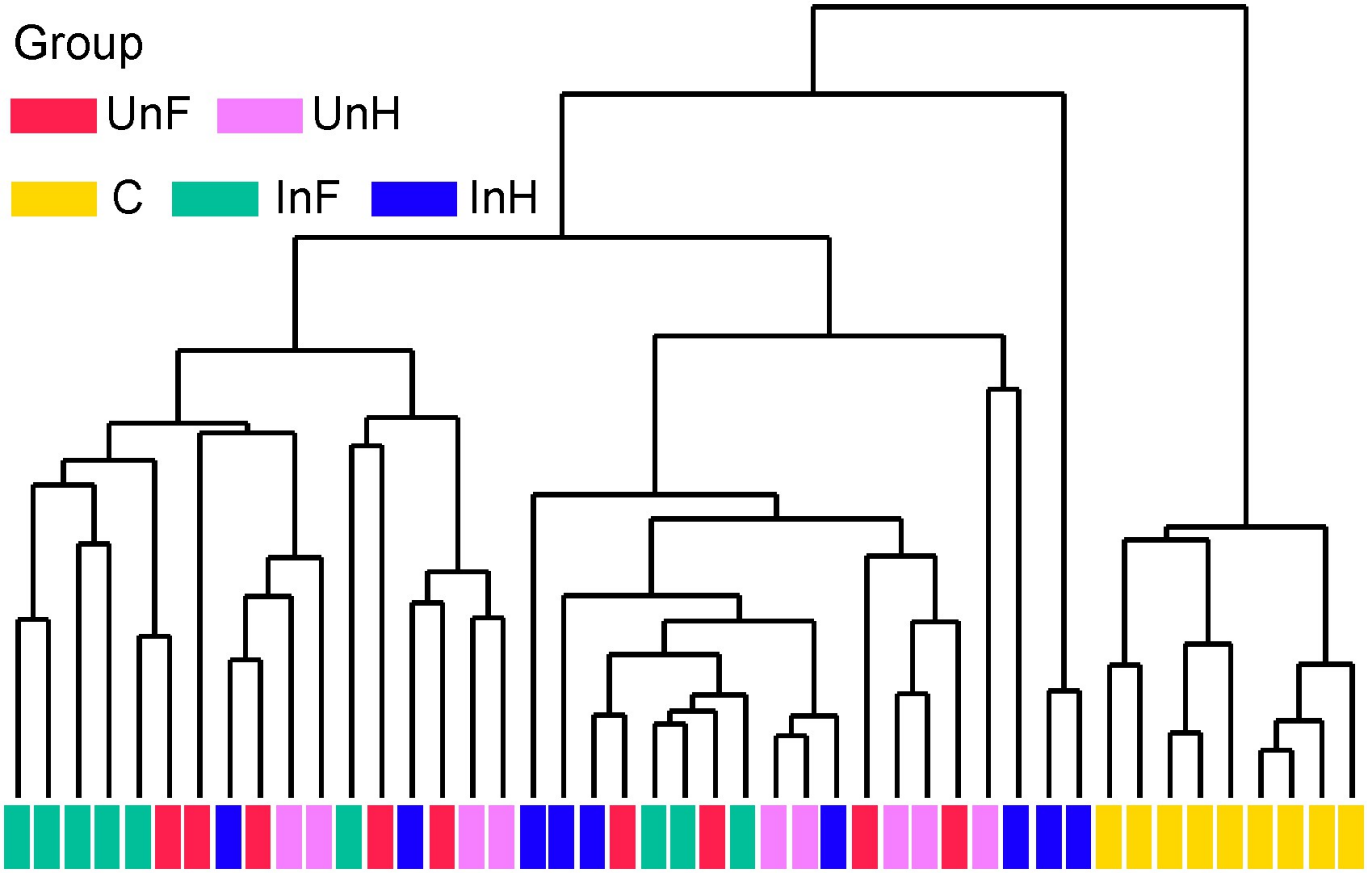
Cluster analysis performed on Bray-Curtis distance matrices of bacterial OTUs using an unweighted pair group mean algorithm. UnF, uninfected with *S*. *acheilognathi* in the foregut of grass carp; UnH, uninfected with *S*. *acheilognathi* in the hindgut of grass carp; C, the surface of *S*. *acheilognathi*; InF, infected with *S*. *acheilognathi* in the foregut of grass carp; InH, infected with *S*. *acheilognathi* in the hindgut of grass carp.

**Fig 5:**
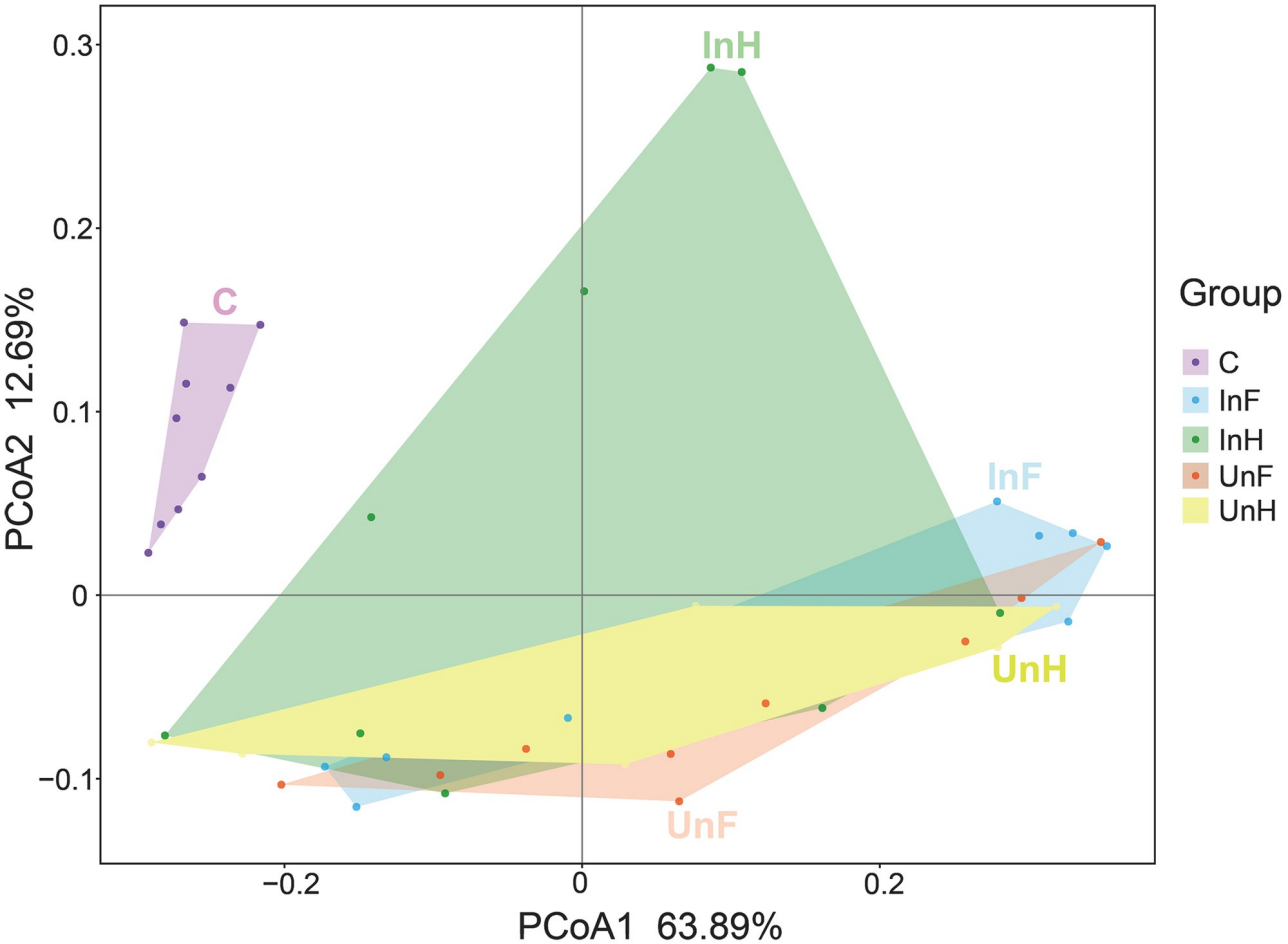
Principal coordinates analysis of microbiota community structures. UnF, uninfected with *S*. *acheilognathi* in the foregut of grass carp; UnH, uninfected with *S*. *acheilognathi* in the hindgut of grass carp; C, the surface of *S*. *acheilognathi*; InF, infected with *S*. *acheilognathi* in the foregut of grass carp; InH, infected with *S*. *acheilognathi* in the hindgut of grass carp.

For the beta diversity, cluster analysis indicated that all samples were divided into two groups (Fig 4), where all the samples from *S*. *acheilognathi* surface clustered into one group, and all samples from the intestine of grass carp clustered into a separate group. PCoA (Fig 5) and PERMANOVA (Table 2) with weighted unifrac distance analyses showed that the C group was separated from the remaining groups (*P<* 0.05 in all cases). InH was significantly different from all groups except UnH. InF was not significantly different from UnF and UnH (*P >* 0.05 in both cases). UnF was not significantly different from UnH (*P* = 0.916).

**Table 2:** PERMANOVA analysis of different groups with the weighted unifrac distance.

	C	UnF	UnH	InF	InH
C		***0*.*001***	***0*.*001***	***0*.*001***	***0*.*001***
UnF	**38.62**		0.916	0.426	***0*.*030***
UnH	**32.96**	0.30		0.352	0.141
InF	**23.01**	0.80	0.98		***0*.*040***
InH	**24.92**	**2.51**	1.78	**2.80**	

Pseudo-F values of the PERMANOVA test are shown in regular font, P-values are italicized, and P-values < 0.05 are bolded. UnF, uninfected with *Schyzocotyle acheilognathi* in the foregut of grass carp; UnH, uninfected with *S*. *acheilognathi* in the hindgut of grass carp; C, the surface of *S*. *acheilognathi*; InF, infected with *S*. *acheilognathi* in the foregut of grass carp; InH, infected with *S*. *acheilognathi* in the hindgut of grass carp.

### Differences in taxonomic abundance among groups

**Fig 6:**
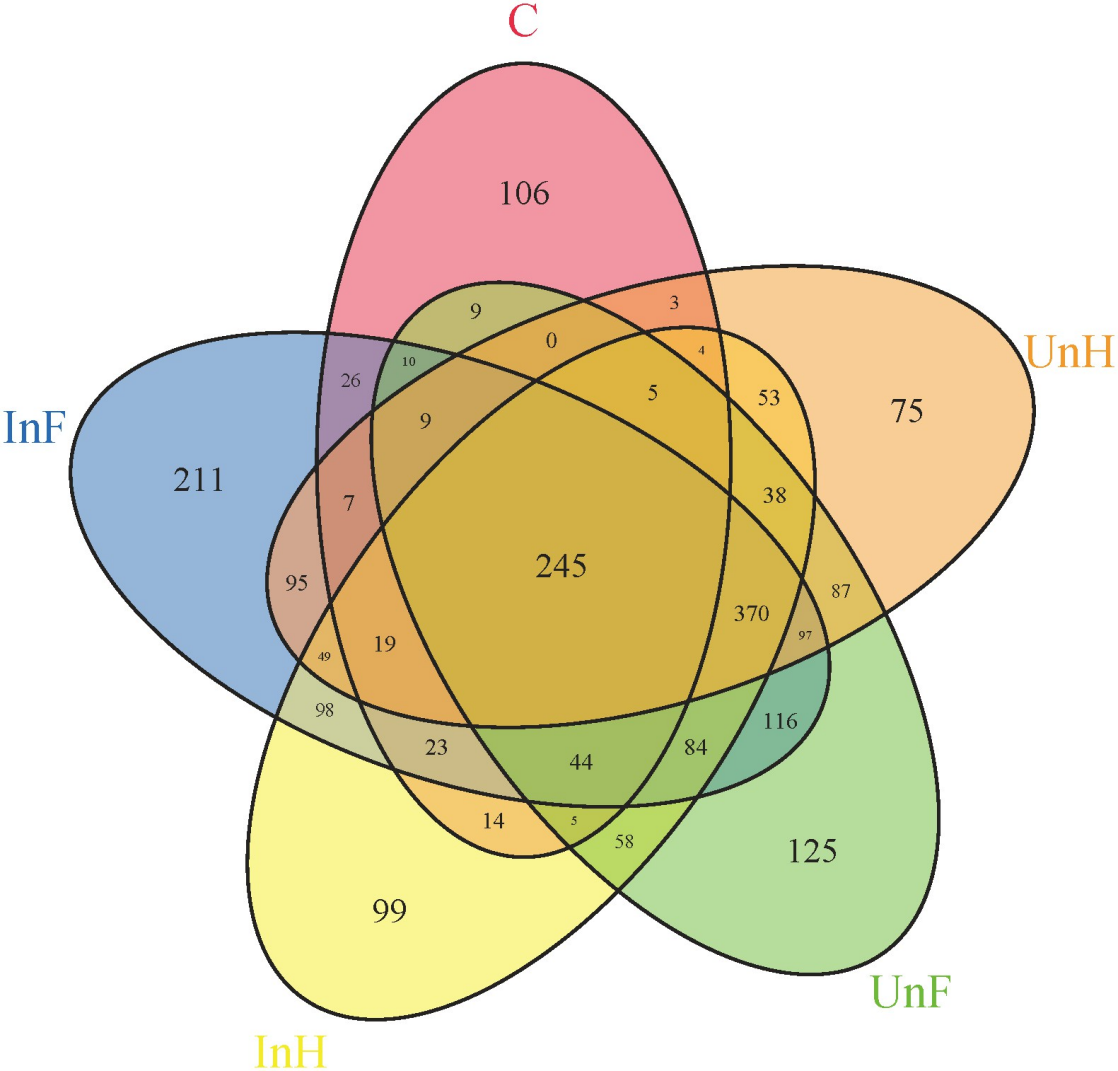
Venn diagram of OTUs in different groups. UnF, uninfected with *Schyzocotyle acheilognathi* in the foregut of grass carp; UnH, uninfected with *S*. *acheilognathi* in the hindgut of grass carp; C, the surface of *S*. *acheilognathi*; InF, infected with *S*. *acheilognathi* in the foregut of grass carp; InH, infected with *S*. *acheilognathi* in the hindgut of grass carp.

Venn diagram showed that total OTUs of UnF, UnH, C, InF and InH groups were 1302, 1156, 529, 1503 and 1208, respectively; unique OUTs of each group were 125, 75, 106, 211 and 99, respectively; and the five groups shared 245 OTUs (Fig 6).

**Fig 7:**
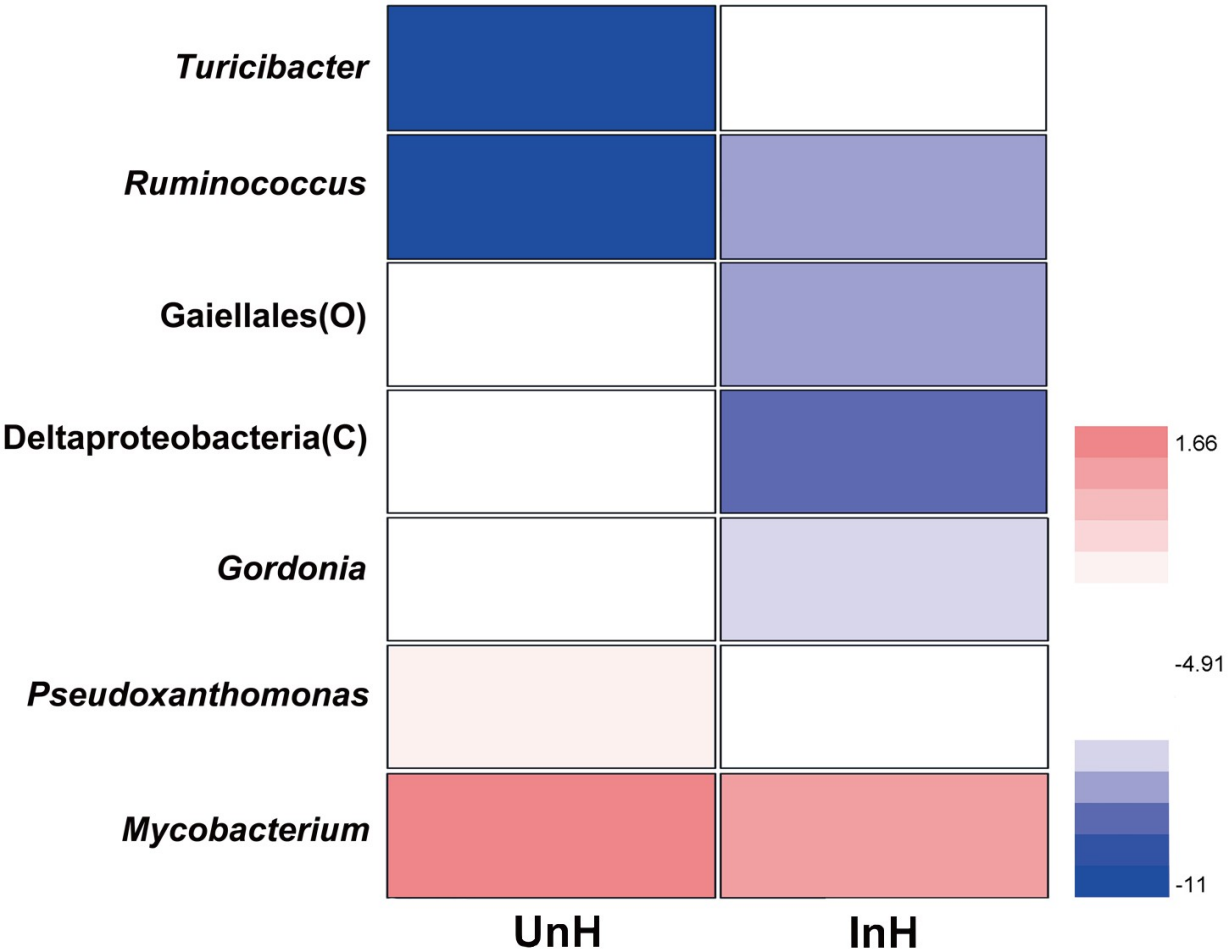
Heatmap of the significant taxa in the hindgut of grass carp. UnH, uninfected with *S*. *acheilognathi* in the hindgut of grass carp; InH, infected with *S*. *acheilognathi* in the hindgut of grass carp.

In the Welch’s t-test of taxonomic abundance at the genus level, there was only one taxon (Betaproteobacteria) exhibited a significant difference between UnF and InF (*P* = 0.049). However, there was seven taxa exhibited significant differences between UnH and InH groups (Fig 7; *P* < 0.05 in all cases; S1 Table).

**Fig 8:**
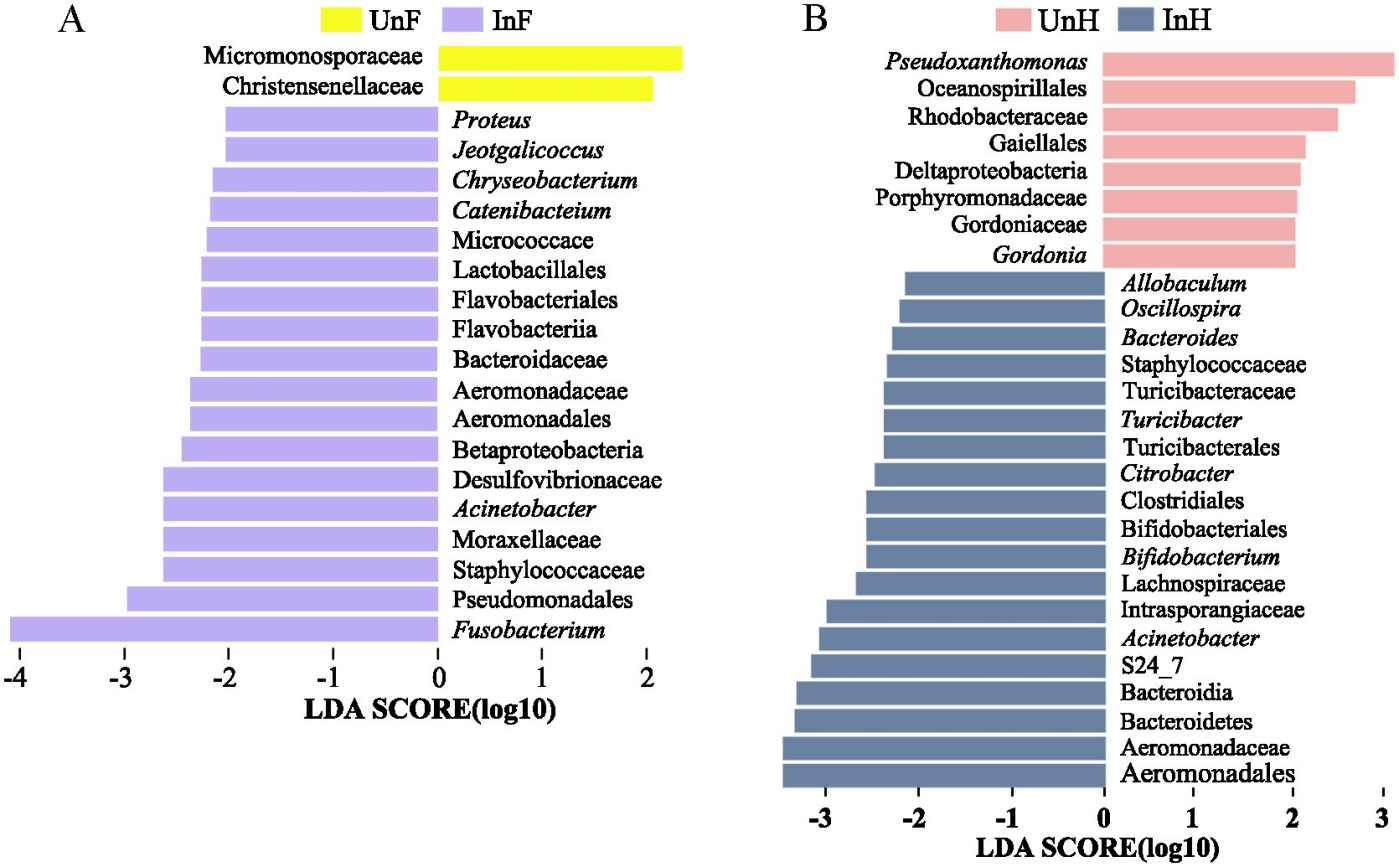
Bacterial taxa with significant differences (LDA score > 2.0) in the relative abundance identified by the Lefse analysis in UnF and InF groups (A), and UnH and InH groups (B). UnF, uninfected with *S*. *acheilognathi* in the foregut of grass carp; UnH, uninfected with *S*. *acheilognathi* in the hindgut of grass carp; InF, infected with *S*. *acheilognathi* in the foregut of grass carp; InH, infected with *S*. *acheilognathi* in the hindgut of grass carp.

Linear discriminant analysis coupled with effect size (Lefse) analysis indicated that there were twenty biomarkers between UnF and InF groups (Fig 8A), and there were twenty-seven biomarkers between UnH and InH groups (Fig 8B) at the genus level. *Acinetobacter*, Aeromonadaceae, Aeromonadales were the shared biomarkers in the gut of grass carp infected with *S*. *acheilognathi*.

### Changes in KEGG pathways

**Fig 9:**
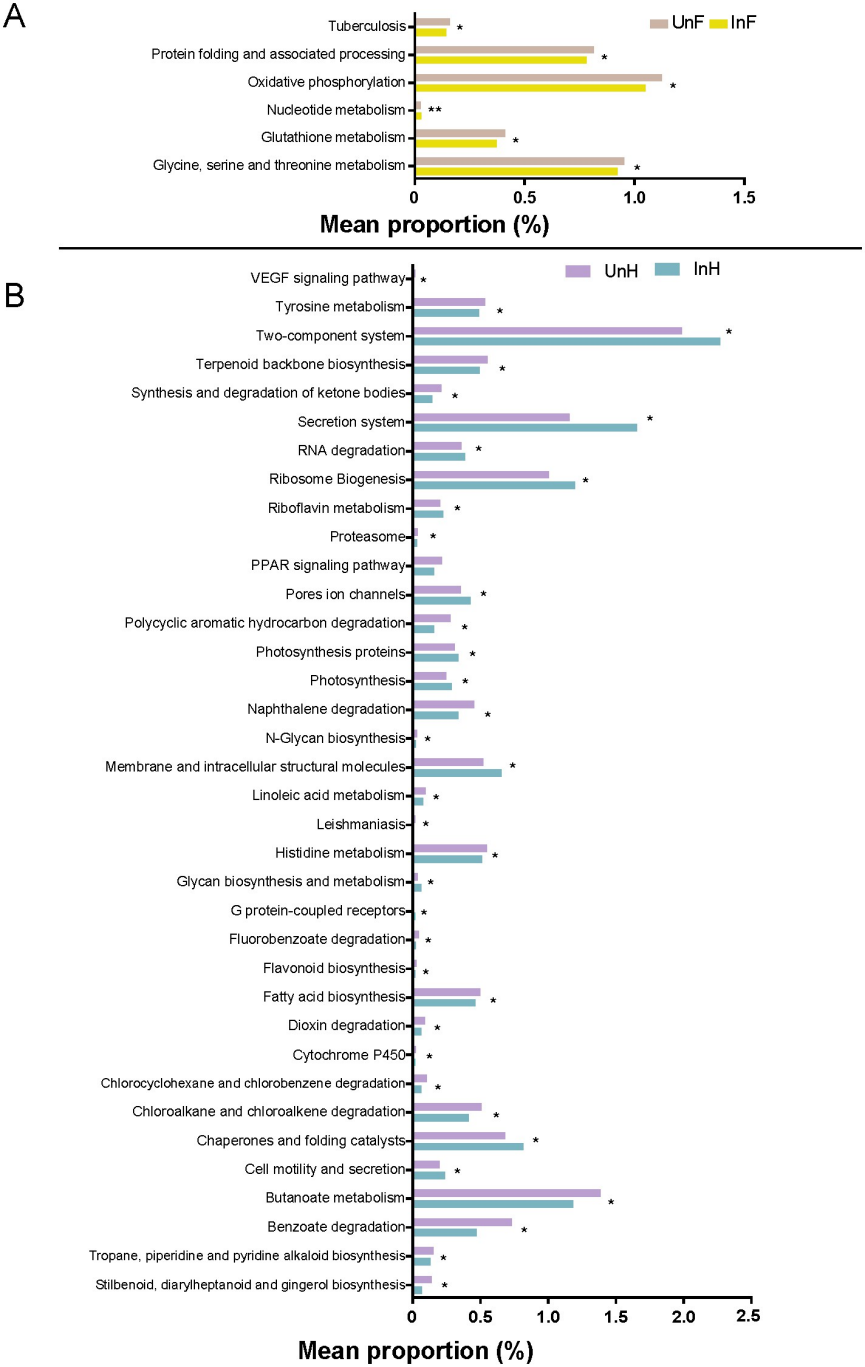
Changes in the KEGG pathways predicted by PICRUST. UnF, uninfected with *S*. *acheilognathi* in the foregut of grass carp; UnH, uninfected with *S*. *acheilognathi* in the hindgut of grass carp; InF, infected with *S*. *acheilognathi* in the foregut of grass carp; InH, infected with *S*. *acheilognathi* in the hindgut of grass carp. *P*-value was calculated using Welch’s t-test for two groups comparison; 0.01 < *P* < 0.05 values are marked with “*” and *P <* 0.01 values are marked with “**”.

The PICRUST prediction revealed that six KEGG pathways in L3 level exhibited significant differences between UnF and InF (Fig 9A), whereas 36 KEGG pathways exhibited significant differences between UnH and InH (Fig 9B). Among these, seven KEGG pathways were related to Xenobiotics Biodegradation and Metabolism (19.4%: 7/36), four KEGG pathways were related to Cellular Processes and Signaling (11.1%: 4/36), three KEGG pathways were related to Lipid Metabolism (8.3%: 3/36), three KEGG pathways were related to Biosynthesis of Other Secondary Metabolites (8.3%: 3/36), three KEGG pathways were related to Folding, Sorting and Degradation (8.3%: 3/36), two KEGG pathways were related to Amino Acid Metabolism (5.6%: 2/36), two KEGG pathways were related to Energy Metabolism (5.6%: 2/36), and two KEGG pathways were related to Signal Transduction (5.6%: 2/36).

### Association between cestode infection and relative abundance of gut microbiota

Pearson correlation analysis showed that wet weight of *S*. *acheilognathi* had a significant positive correlation (*P* < 0.05 in all cases) with the relative abundance of *Acinetobacter*, Betaproteobacteria [C], Bacteroidaceae, Desulfovibrionaceae, *Chryseobacterium*, *Catenibacterium*, *Turicibacter*, Sphingomonadales[O], Christensenellaceae (negative) in the foregut (0.48 ≤ *r* ≤ 0.58); and there were five taxa (*Turicibacter*, *Ruminococcus*, *Proteus*, *Facklamia*, *Oscillospira*) having a significant positive correlation (*P* < 0.05 in all cases) with the tapeworm wet weight (0.48 ≤ *r* ≤ 0.63, *P* < 0.05 in all cases), six taxa (*Gordonia*, *Pseudoxanthomonas*, Gaiellales [O], *Mycobacterium*, Deltaproteobacteria [C], *Tessaracoccus*) having a significant negative correlation with the tapeworm wet weight (0.47 ≤ |*r*| ≤ 0.58, *P* < 0.05 in all cases) in the hindgut (Table 3). *Turicibacter* was the only one bacterial taxon showing a significant positive correlation with the tapeworm wet weight in the foregut and hindgut.

**Table 3:** Correlation coefficient analysis between tapeworm wet weight and abundance of each bacterium in the intestine of grass carp (*Ctenopharyngodon idella*).

	Taxon	*r*	*P*
**Foregut**	*Acinetobacter*	0.58	0.011
	Betaproteobacteria[C]	0.58	0.012
	Bacteroidaceae	0.51	0.029
	Desulfovibrionaceae	0.49	0.038
	*Chryseobacterium*	0.49	0.038
	*Catenibacterium*	0.49	0.041
	*Turicibacter*	0.48	0.044
	Sphingomonadales[O]	0.47	0.049
	Christensenellaceae	-0.51	0.031
**Hindgut**	*Turicibacter*	0.63	0.005
	*Ruminococcus*	0.54	0.022
	*Proteus*	0.50	0.035
	*Facklamia*	0.48	0.042
	*Oscillospira*	0.48	0.043
	*Gordonia*	–0.58	0.013
	*Pseudoxanthomonas*	–0.53	0.024
	Gaiellales[O]	–0.53	0.025
	*Mycobacterium*	–0.50	0.036
	Deltaproteobacteria[C]	–0.48	0.045
	*Tessaracoccus*	–0.47	0.049

### Effects of tapeworm infection on the growth of grass carp

**Fig 10:**
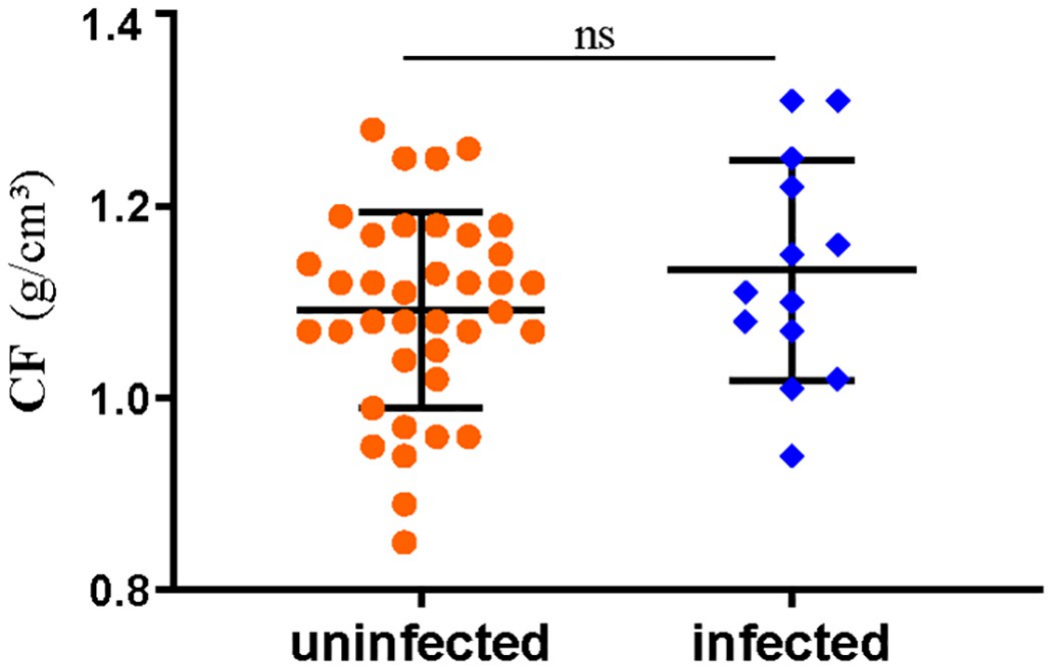
The condition factor of grass carp (uninfected group, without *S*. *acheilognathi* infection; infected group: Infection with *S*. *acheilognathi*; ns, no significance).

The prevalence of *S*. *acheilognathi* infection among the experimental grass carp specimens was 25.49% (13 infected among 51 in total). The cestode wet weight (CWW) in infected fish was recorded (S2 Table); we did not record the cestode weight in the three fish specimens that were infected with only one tapeworm The body length (L) and weight (W) of each fish (S2 Table) were measured to calculate the condition factor (CF). The condition factor reflects the physiological state of the fish [38], and it was used to assess the impact of *S*. *acheilognathi* on the growth of grass carp. The CF was calculated as follows: CF = (weight / body length^3^) * 100 [38]. The results showed that there was no significant difference in the CF of grass carp between the infected group and the uninfected group (*t*_(49)_ = –1.536, *P* = 0.13 > 0.05) (Fig 10).

### Effects of tapeworm infection on the intestinal tissue structure

**Fig 11:**
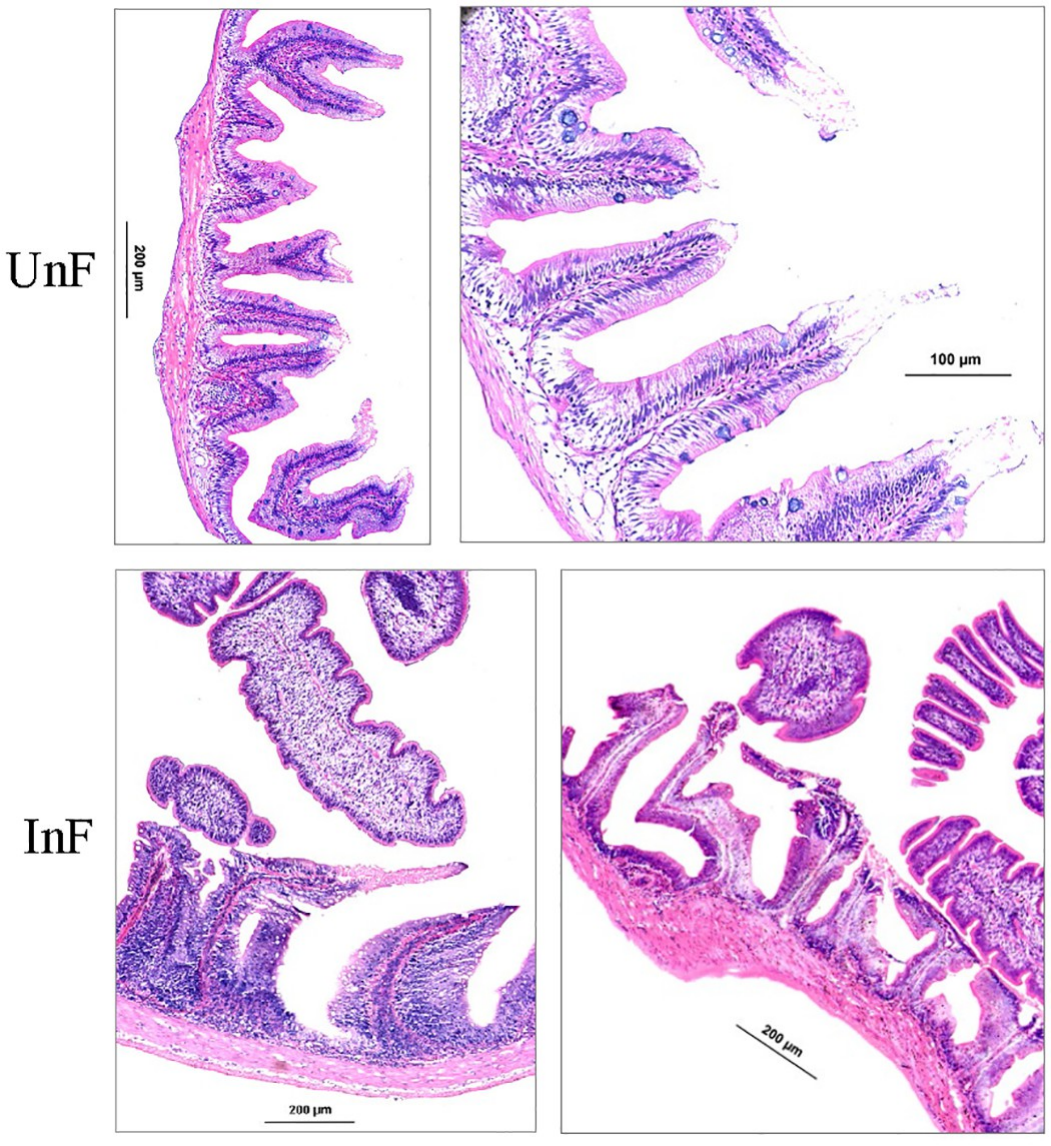
Tissue sections of the foregut in grass carp. UnF, uninfected with *S*. *acheilognathi* in the foregut of grass carp; InF, infected with *S*. *acheilognathi* in the foregut of grass carp.

There were tapeworm proglottides and scoleces in the foregut histological sections in the infected grass carp, and tapeworm infection caused serious damage to the foregut villi of grass carp, and the epithelial cells were severely degenerated, necrotic, and exfoliated (Fig 11).

### Effects of tapeworm infection on the relative expression of immune-related genes in the intestinal mucosa of grass carp

**Fig 12:**
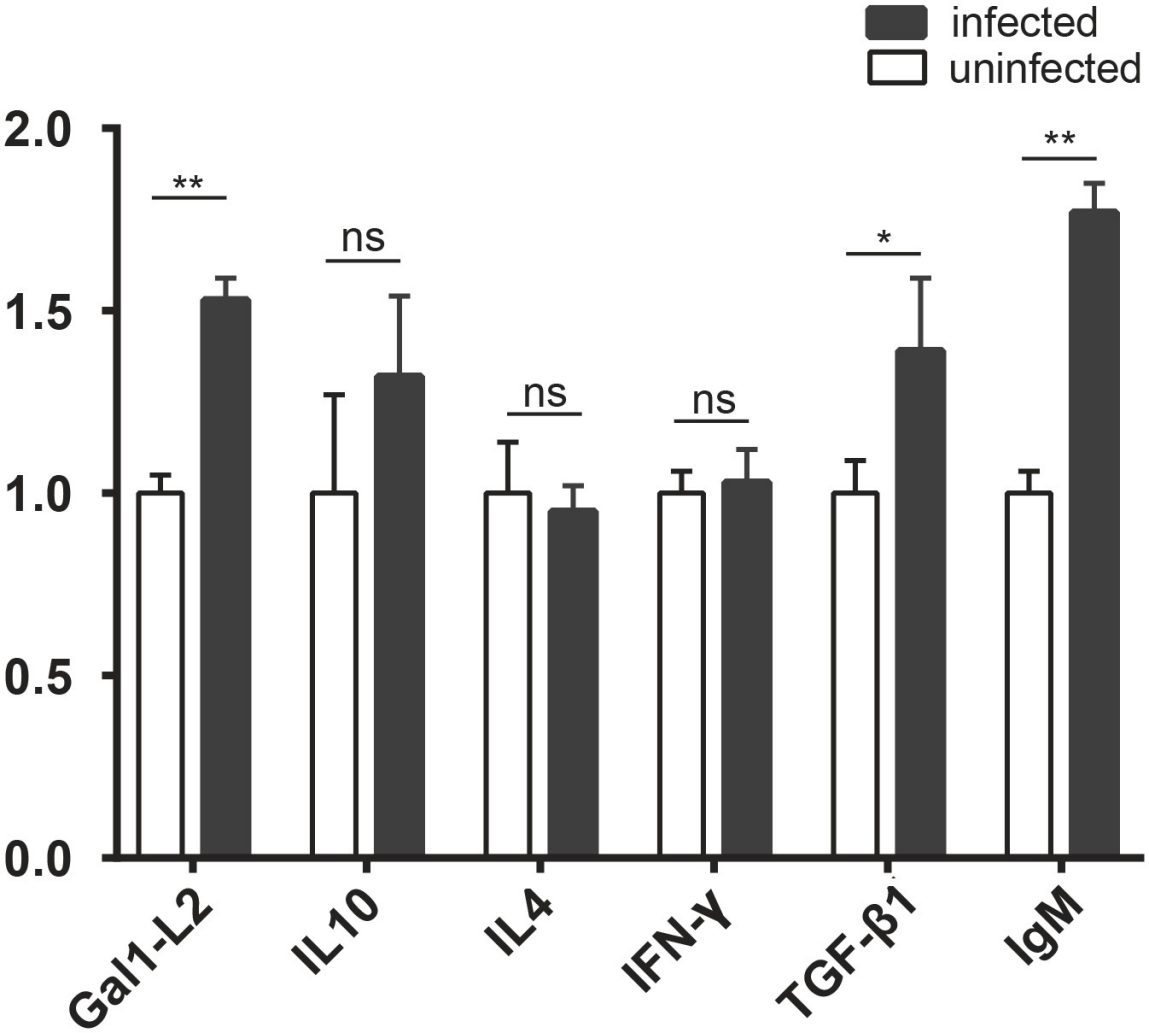
Relative expression of immune-related genes in the intestinal mucosa of grass carp. Uninfected group, without *S*. *acheilognathi* infection; infected group, infection with *S*. *acheilognathi*; ns, no significance; “**”, *P* < 0.01; “*”, 0.05 < *P* < 0.01.

We measured the mRNA expression levels of *Gal1*-*L2*, *IL*-*4*, *IL*-*10*, *TGF*-*β1*, *IFN*-*γ*, and *IgM* genes (Fig 12). The relative expression of *Gal1*-*L2*, *TGF*-*β1*, and *IgM* significantly differed between uninfected and infected groups (*t*_3,3_ = 12.649, *P* = 0.000 < 0.05; *t*_3,3_ = 2.973, *P* = 0.041 < 0.05; *t*_3,3_ = 14.916, *P* = 0.000 < 0.05, respectively). However, the relative expression levels of *IL*-*4*, *IL*-*10*, *IFN*-*γ* did not significantly difference between uninfected and infected groups (*t*_3,3_ = –0.537, *P* = 0.620 > 0.05; *t*_3,3_ = 1.597, *P* = 0.186 > 0.05; *t*_3,3_ = 0.460, *P* = 0.669 > 0.05, respectively).

## Discussion

Tripartite interaction among helminths, intestinal microbiota and host has been studied mainly in mammals, but never in fish. In our study, we explored the effects of tapeworm *S*. *acheilognathi* infection on the growth, immune system and gut microbiota of grass carp. Intestinal helminth infection often alters the composition of gut microbiota in mammal animals, but rarely affects the alpha diversity [1]. In line with the results of previous studies, *S*. *acheilognathi* infection also did not significantly affect the microbial alpha diversity in grass carp, but it did change the microbial composition. However, the effects on composition were relatively small, observable only on the genus or family levels. This is similar to the effects of *Hymenolepis diminuta* infection on the fecal microbiota of rats [39].

*Schyzocotyle acheilognathi* infection significantly increased the relative abundance of *Turicibacter*, but this is in disagreement with a previous observation that *H*. *diminuta* infection caused significant changes in the composition of cecal microbiota, most significant of which was the decrease of *Turicibacter* [6]. *Turicibacter* spp. are spore-producing anaerobic bacteria that inhabit the intestines of humans, pigs, cats and rabbits [40–42]. *Turicibacter* is closely related to the regulation of serotonin in the intestinal tract: increased levels of serotonin in the intestinal lumen increase the abundance of *Turicibacter* in the intestine. Guinea pigs infected with the parasite nematode *Trichostrongylus colubriformis* (both primary and secondary infection) had significantly increased serotonin level in the intestinal mucosa [43]. *Turicibacter* can regulate the lipid and steroid metabolism of the host, reduce the content of triglycerides in the serum, thereby affecting the physiology of the host [44]. In our study, PICUST prediction also showed that *S*. *acheilognathi* infection significantly altered three lipid metabolism-related pathways in the hindgut of grass carp. These results indicated that helminth-modified intestinal bacteria composition, most notably increased relative abundance of *Turicibacter*, may affect the lipid metabolism in grass carp.

Cestodes lack an alimentary canal and the structural basis of membrane digestion in tapeworms are the microtriches on the tegument surface [29]. Several morphological forms of symbiotic bacteria and nanobacteria (bacteria with sizes less than micron) were found in tapeworms [45–48], but the identity and functions of these bacterial taxa are unknown. Therefore, 16S sequencing technology was used to study the microbiota on the surface of tapeworm for the first time in our study. Fusobacteria, Proteobacteria, Bacteroidetes and Tenericutes formed the dominant bacterial taxa (relative abundance > 1%) at phylum level on the cestodes’ surface. Compared with the intestinal microbiota of grass carp, there was a higher relative abundance of Bacteroidetes and Tenericutes, and lesser of Actinobacteria and Firmicutes on the surface of *S*. *acheilognathi*. At genus level, majority of the bacterial taxa on the surface of *S*. *acheilognathi* was *Fusobacterium*, which differed from the intestine of grass carp, in which *Cetobacterium* and *Rhodobacter* were dominant. Furthermore, microbial alpha diversity on cestodes’ surface was significantly lower than in the intestine, and the beta diversity analysis indicated that bacterial communities significantly differed between the cestodes’ surface and intestine. These analyses indicated that *S*. *acheilognathi* habored special microbiota, differing from the intestine of grass carp. This phenomenon has also been observed between the nematode *T*. *muris* and its host: the parasitic *T*. *muris* acquired a distinct intestinal microbiota from its host, which was required for its fitness [4]. The strategy could promote successful chronic nematode infection. The function of the special bacterial taxa on the cestodes’ surface may differ from *T*. *muris*. *Schyzocotyle acheilognathi* parasitizes in the foregut of grass carp, but food in the foregut is mainly macromolecules, while tapeworms have no digestive tract and rely on the microtriches to absorb nutrients. Macromolecular substances cannot be directly absorbed, and the symbiotic microbiota on the tapeworm’s body surface may also play a role in decomposing nutrients for tapeworms.

Parasites often exhibit negative effects on their hosts [49–51], and *S*. *acheilognathi* infection had also been reported to negatively affect the growth of the host [52, 53]. However, we obtained a different result: *S*. *acheilognathi* infection had no effect on grass carp growth. The result is consisted with the findings of Henriksen et al. [54], who also found no direct negative associations between parasite abundance and fish growth found in his study. The relationship between parasite abundance and growth was linearly positive for the low-impact *Crepidostomum* sp. [54]. It seems to indicate that *S*. *acheilognathi* is a low-impact parasite for grass carp. For fish, individual growth rates are positively correlated with food consumption [55], and elevated consumption rates increase the exposure to trophically transmitted parasites. Therefore, fish that eat more, grow faster and have more parasites. However, three-spined sticklebacks infected with the large-sized cestode *S*. *solidus* were able to sustain high growth rates if access to food was not limited [56]. The grass carp used in our study had plenty of food, which also might be one a reason why *S*. *acheilognathi* infection had no effect on the growth of grass carp.

*Schyzocotyle acheilognathi* has received considerable attention for its pathogenic effects, which include blocking of the intestine, inflammation in the intestine, and perforation of the intestinal wall [30, 57]. According to the intestinal pathological tissue section, we found that *S*. *acheilognathi* infection with grass carp caused severe damage to intestinal epithelial cells and caused intestinal local mucosal immunity, which is in agreement with previous research results.

*Schyzocotyle acheilognathi* infection significantly upregulated the expressions of mucosal immune-related genes *Gal1*-*L2*, *TGF*-*β1* and *IgM* in the foregut, but not *IL*-*4*, *IL*-*10* and *IFN*−*γ*. Galectin-1(*Gal1*) exists in many organisms and it is widely distributed in mucosal tissues. It has a variety of immunomodulatory functions, including anti-inflammatory [58], pathogen recognition, and resistance to bacterial and viral infections [59]. In the present study, the expression of *Gal1* in the foregut of grass carp was upregulated due to the *S*. *acheilognathi* infection, which was consistent with the results of Hoorens (2011) [60]. *Gal1* expression was up-regulated in both primary and secondary infection with *Ostertagia ostertagi* in cattle, suggesting that *Gal1* was involved in protective immunity. The activation of the host’s protective immune response against intestinal helminth infection requires the participation of multiple cytokines. *TGF*-*β* was upregulated in response to *Trichinella spiralis* infection; it activated the Th17 immune response and stimulated intestinal contraction, thus promoting *T*. *spiralis* excretion [61]. In this study, *TGF*-*β1* was upregulated by the *S*. *acheilognathi* infection, suggesting that *TGF*-*β1* plays an important role in the host resistance to helminth infection. *IgM* is the primary immunoglobulin of bony fish, which plays an important role in the humoral immunity of fish [62]. *IgM* was highly expressed in mucosa-related tissues (such as intestine, gills and skin) of fish [63–65]. Parasite-specific *IgM* has a significant anti-parasitic activity, which plays an important role in the protective immune response to *Plasmodium chabaudi* in mice during the asexual erythrocytic phase, delaying parasitemia and reducing host mortality [66]. *Schyzocotyle acheilognathi* infection upregulated the expression of *IgM* in the intestine of grass carp, which was similar to the results in Myxosporea. A significant increase of the *IgM* expression was detected only in the gilthead sea bream (*Sparus aurata*) infected with *Enteromyxum leei*, but only very late after the exposure [67].

## Conclusion

Effects of helminth infection on the intestinal microbiota, growth and immune reactions of the host were explored under laboratory conditions in fish. *Schyzocotyle acheilognathi* infection altered the composition of intestinal microbiota, but it did not affect the microbial alpha diversity. Helminth-modified intestinal bacteria composition, most notably increased relative abundance of *Turicibacter*, may affect the lipid metabolism in grass carp. Finally, although *S*. *acheilognathi* infection activated intestinal local mucosal immunity of the host, it had no effect on the growth of grass carp.

## Supporting information

S1 Table Bacterial taxa with a significant difference in the relative abundance between UnH and InH. (XLSX)

S2 Table Body length and weight measurement of grass carp and record of *Schyzocotyle acheilognathi* infection in grass carp intestine. (XLSX)
